# Meat Quality of Dairy and Dairy × Beef Steers Reared in Two Production Systems Based on Forages and Semi-Natural Pastures

**DOI:** 10.3390/ani15081081

**Published:** 2025-04-08

**Authors:** Qasim Mashood, Anna Hessle, Viktoria Olsson, Margrethe Therkildsen, Søren Krogh Jensen, Katarina Arvidsson Segerkvist

**Affiliations:** 1 Department of Applied Animal Science and Welfare, The Swedish University of Agricultural Sciences, 532 23 Skara, Sweden; anna.hessle@slu.se (A.H.); katarina.segerkvist@slu.se (K.A.S.); 2 Department of Food and Meal Science, Kristianstad University, 291 88 Kristianstad, Sweden; viktoria.olsson@hkr.se; 3 Department of Food Science, Aarhus University, Agro Food Park, 8200 Aarhus, Denmark; margrethe.therkildsen@food.au.dk; 4 Department of Animal and Veterinary Sciences, Aarhus University, Blichers Allé 20, 8830 Tjele, Denmark; skj@anivet.au.dk

**Keywords:** biodiversity, semi-natural grasslands, pasture-based, beef, technological traits, sensory attributes, fatty acids

## Abstract

To safeguard an agricultural landscape with high biodiversity, livestock grazing on semi-natural pastures is crucial, and steers are well suited to such production systems. This study compared meat quality, including technological traits, sensory attributes, and fatty acid composition, of purebred dairy steers (D) and dairy × beef crossbreed steers (C), reared in two distinct production systems. Sixty-four steers (thirty-two per breed type) were included. Half were kept in a production system that had relatively high feed intensity (H), with one grazing summer on semi-natural pastures and slaughtered at 21 months of age. The other half were kept in a production system that had low feed intensity (L), with two grazing summers on semi-natural pastures and slaughtered at 28 months. Colour, water holding capacity, Warner–Bratzler shear force, sensory attributes, and fatty acid profiles were measured on the *Musculus longissimus lumborum*. Meat from L steers with two grazing seasons was darker (*p =* 0.003) and contained a higher proportion of unsaturated fatty acids (*p =* 0.006) than meat from more intensively reared H steers. Meat from C steers was perceived as having a coarser fibre structure (*p =* 0.022) with an acidic odour (*p =* 0.040) compared to D steers. Additionally, cooked meat from L steers was evaluated as having a pinker appearance by an analytical sensory panel (*p =* 0.008). In summary, breed type and production system had no major effect on technological and sensory attributes for forage and pasture-fed steers, but fatty acid composition was improved with more unsaturated lipids in meat from L steers.

## Simple Summary

This study investigated how breed type and production system affect meat quality in steers raised on semi-natural pastures, which is essential for biodiversity. This research compared purebred dairy steers (D) and dairy × beef crossbreeds (C), each raised in either a high-intensity system (H) with one grazing season or a low-intensity system (L) with two grazing seasons. Meat from L steers was darker and had higher levels of beneficial unsaturated and omega-3 fatty acids. C steers showed slightly coarser muscle texture and a more acidic odour but had higher omega-3 levels than D steers. Meat quality traits such as tenderness and water-holding capacity were primarily unaffected by breed or system. Both breed types and systems produced comparable meat quality, but the L system offered nutritional benefits. These findings support the use of extensive grazing on biodiverse pastures as a sustainable beef production strategy that maintains meat quality while supporting biodiversity.

## 1. Introduction

The global decrease in biodiversity due to habitat loss is the prime outcome of land usage [1,2]. A rapid decrease in the area of semi-natural pastures has taken place all over Europe [3], causing negative effects for grassland specialist flora [4]. Semi-natural pastures are grasslands that have developed through long-term traditional grazing or mowing practices without significant alteration by modern agriculture, such as ploughing, reseeding, or fertilisation. These pastures are typically rich in biodiversity, supporting a variety of plant and animal species [5]. While Sweden is facing acute threats of under-grazing and abandonment of pastures, 340,000 hectares of semi-natural grasslands in this country are still grazed on and are therefore valuable for biodiversity [6]. Safeguarding agricultural landscapes through traditional pasture-based livestock production is a necessary tool to ensure high biodiversity [7]. The production value of these semi-natural pastures is low [8], but the management of these pastures through livestock grazing [9] provides ecosystem services and creates habitats for many endangered species [4,10]. To promote grazing on these lands, agri-environmental payments in Sweden and many other European countries favour extensive beef production [11,12]. It is also possible to use grazing of semi-natural pastures as an added value when marketing beef, rendering a higher price with increased consumer demand [13]. Thus, livestock grazing with dairy off-spring on semi-natural pastures provides synergism of both sustainable food production (meat and milk products) and maintenance of biodiversity [14].

Beef production has a critical impact on the environment through the emission of greenhouse gases [15]. A review concluded that beef production from dual-purpose cows or dairy cows inseminated with semen from beef breed bulls shows the potential to reduce the climate impacts of beef compared to suckler-based beef [16]. Numerous studies conducted worldwide have examined the effects of crossbreeding between dairy and beef breeds, concluding that it successfully increases both the quantity and quality of beef [17,18,19]. However, a significant limitation of these studies was the sole use of intensive rearing systems, rather than incorporating conservation grazing (a practice which integrates livestock production with the maintenance of biodiversity) into the production of high-quality meat. Comparably, some research has evaluated the potential of raising crossbred cattle (dairy × beef) in a large-scale production system with semi-natural grassland [20,21], demonstrating their economic potential for farmers together with the conservation of biological values [22]. A knowledge gap that requires investigation is how an extensive production system with semi-natural grassland affects various meat quality traits in crossbred (dairy × beef) animals [23,24], particularly in steers, which are preferable over intact bulls because of their superior grazing ability and better temperament. A recent study on crossbred heifers (dairy × beef) grazed on biodiverse semi-natural pastures uncovered the possibility of producing high-quality, heavier carcasses with more conformation but less fatness and marbling scores [21,25]. Despite evidence that crossbreeding enhances meat quality, little is known about how grazing on semi-natural grasslands influences key meat quality parameters in steers. Grazing on semi-natural pastures has been associated with improved omega-3 fatty acid content and a more favourable balance of saturated and unsaturated fats compared to grain-based feeding systems [26]. Additionally, differences in forage intake and movement patterns may influence muscle development, affecting meat texture [27]. Understanding these effects is crucial for developing sustainable beef production systems that align with both economic and environmental goals, while also meeting consumer expectations for high-quality meat. Given the potential benefits of extensive grazing systems, we hypothesise that dairy × beef crossbreed steers will exhibit superior meat quality characteristics compared to purebred dairy steers. Additionally, we expect that grazing on biodiverse semi-natural pastures will influence fatty acid composition, enhancing the nutritional value of the meat while maintaining desirable sensory properties.

Disregarding breed, several other factors, including age at slaughter, production system, body weight, gender, pre-slaughter handling, and meat ageing, affect the carcass quality, meat quality, and sensory properties of meat [28,29]. The aim of this study was to investigate the technological characteristics, sensory properties, and fatty acid composition of meat from steers originating from dairy cows and compare purebred dairy steers with dairy × beef crossbreeds reared in two different production systems. Both production groups were grazed on semi-natural pastures, but the indoor feed intensity and slaughter age differed. The production results were reported previously in [20].

## 2. Materials and Methods

### 2.1. Animals and Rearing

This study had a 2 × 2 factorial design involving two production systems and two breed types: dairy breed and crossbreed. The rearing of the experimental cattle was conducted at the Götala Beef and Lamb Research Centre, Swedish University of Agricultural Sciences (SLU), Skara, in south-western Sweden.

In total, 64 steers, comprising the two breed types (dairy, D, and dairy × beef, C), were followed from weaning (3–4 months of age) to slaughter, where 32 steers were of dairy breed (D; 12 Swedish Red and 20 Swedish Holstein), and the other 32 were crossbreeds between dairy and beef breed (C; 12 Swedish Red × Charolais and 20 Swedish Holstein × Charolais). During indoor periods, the steers were kept and fed in groups of four in pens with fully slatted floors. The steers were continuously introduced into the experiment according to birth date, with pairs of calves from the two breed types introduced simultaneously. Hence, the age of individual calves and calves in pens was matched pairwise across breeds. Steers in a pair of pens, i.e., four D steers and four C steers, were slaughtered at the same time, when the average age reached the target age. Steers were slaughtered at a commercial abattoir situated at a 30 min drive from the farm, where they were stunned with a captive bolt pistol and slaughtered by exsanguination. The carcass traits are presented in Table 1, and a complete description of the rearing and carcass measurements is available in [20]. Half of the animals from both breed types were subjected to a production system that had moderately high feed intensity by receiving a total mixed ration (TMR) based of grass/clover silage and rolled barley, complemented with rolled peas and soybean meal at young ages. The protein and energy concentration of the feed rations were adjusted to ensure a live weight gain of 0.9 kg/day, with a more nutrient-dense TMR, primarily based on early-cut, high-quality silage, during their initial indoor period. For the H steers, this was followed by nearly five months of grazing on semi-natural pastures, starting when they were 10–12 months old. After returning indoors, they were again fed early-cut forage and subsequently slaughtered at 21 months of age after their second indoor period. In contrast, the L group of animals from both breed types was managed in a production system that had a low feed intensity during their first indoor period, receiving a TMR with less concentrate and based on late-cut silage. They were turned out to semi-natural pastures at 6–9 months of age for their first grazing period, followed by a second indoor period, when they were fed late-cut forage, leading to restricted weight gain, and then kept on semi-natural pastures for a second grazing period of five months. During their third winter, their rearing was finished indoors on early-cut forage, and they were slaughtered at 28 months of age. All silages used contained 90–95% grass species (*Lolium perenne*, *Festuca pratensis*, *Phleum pratense*, and *Festulolium arundinacea* L.) and 5–10% clover species (*Trifolium repens*, *Trifolium pratense*). Both groups grazed on semi-natural pastures with a similar botanical composition, consisting mainly of *Deschampsia cespitosa* (tufted hairgrass) and *Festuca rubra* (red fescue). In addition, *F. ovina* (sheep’s fescue), *D. flexuosa* (wavy hairgrass), *Nardus stricta* (matgrass), *D. cespitosa*, *F. rubra*, *D. cespitosa*, Cyperaceae (sedges/rushes), and several herb species were present. Feed values and the chemical composition of experimental diets and pastures can be seen in [20]. Three steers were lost due to death or disease, and data from these animals were excluded from further analyses.

### 2.2. Sampling

The pH and temperature values in all carcasses were measured 24 h after slaughter by inserting a probe (Seven2Go pro, Metler Toledo, Schwerzenbach, Switzerland) into the *Musculus longissimus lumborum* (LL). The pH metre was calibrated in buffers with pH 4.01 and 7.00 adjusted to 5 °C. After slaughter, carcasses were stored at a refrigeration temperature of 4 °C until sampling. Forty-eight hours postmortem, LL was sampled and divided into parts to provide samples for different analyses. The analyses of colour, cooking loss, Warner–Bratzler shear force (WBSF), and sensory properties were conducted after a seven-day ageing period. The samples were vacuum-packed and aged at 4 °C for seven days, after which they were frozen at −20 °C until further analyses.

### 2.3. Technological Characteristics

The meat samples were thawed overnight at 4 °C. A fresh cut was made, and samples were allowed to bloom for 1 h at room temperature. Colour measurements were performed over the LL muscle cross section area using Minolta CM-600d (Minolta, Osaka, Japan) calibrated against a white tile (L* = 93.9, a* = 0.3155, b* = 0.3319) according to the manufacturer instruction manual. The aperture was 8 mm, and an illuminant D65 and a 10^°^ standard observer were used. The three variables L* (lightness), a* (redness), and b* (yellowness) were registered from five different locations of meat samples to obtain a representative average value of meat colour. Cooking loss was determined by cutting meat samples into the dimensions of 8 × 5 × 4 cm, which were weighed, packaged in vacuumed plastic bags, and placed in a water bath at a temperature of 62 °C, then cooked until the internal temperature reached 62 °C. After cooking, samples were cooled in a 4 °C cold-water bath for 1 h, dried, and weighed again. Cooking loss was calculated as the difference between sample weights before and after heat treatment [30]. After weighing, samples were trimmed into slices of 10 mm on all four sides along the muscle fibre direction (eight replicates/meat sample). Texture was measured at room temperature as max WBSF [31], using a Stable Micro System texture analyser (Godalming, UK) equipped with a Warner–Bratzler shear blade with a rectangular hole, 11 mm wide, 15 mm high, and a blade thickness of 1.2 mm. The maximum shear force sheared across the fibre direction was recorded at a test speed of 50 mm/min according to the procedure described by [30]. The data were processed using exponent software, and the means of the WBSF of eight replicates/meat sample are presented.

### 2.4. Sensory Properties

After thawing at room temperature, the LL samples intended for sensory analysis were cooked in a sous vide manner to an internal temperature of 60 °C. The meat was cooled, trimmed, and sliced in 5 mm thick slices that were placed in three-digit-coded Petri dishes and reheated to eating temperature directly before serving in a sensory laboratory designed according to SS-EN ISO 8589:2010 [32]. A selected and trained analytical panel (ISO 3972:2011 [33], SS-EN ISO 8586:2014 [34]) consisting of six assessors evaluated the sensory attributes using descriptive analyses that are categorised in Table 2 and the software EyeQuestion (5.12.12). Samples were served in a randomised order and in triplicate, meaning that the panel tested meat from the same steers three times. The intensities of selected properties describing the odour, appearance, texture, basic taste, and flavour of the meat were registered on a line scale ranging from 0 to 100 with indented anchors placed at 10 and 90. Wheat wafers and water were provided for cleansing the palate (Table 2).

### 2.5. Fatty Acid Analysis

Lipid was extracted by homogenising the sample with a mixture of chloroform and methanol, as described by [35], and the fatty acid composition was analysed using the improved method, where fatty acids were esterified with methanol in sodium hydroxide and catalysed by borontrifluride. Gas chromatography with a flame ionisation detector (GC-FID, carrier gas helium HP 6890) on a CP-sil 88 column (50 m, ID 0.25 mm, film 0.20 lm Chrompack, Sao Paulo, Brazil) was used to analyse methyl esters as described by [36]. The methyl esters were identified by comparing the retention times of fatty acid methyl ester (FAME) standards, and fatty acids were divided into the following major categories: saturated fatty acids (SFAs), unsaturated fatty acids (UFAs), including monounsaturated fatty acids (MUFAs), and polyunsaturated fatty acids (PUFAs).

### 2.6. Statistical Analysis

Data were statistically analysed using the MIXED procedure of the Statistical Analysis Software (SAS, Version 9.4). Meat quality attributes were recorded for the individual animal nested within each pen. Since the data were quantitatively gathered to assess trait intensity, the same statistical procedure was applied to all analysed variables. To account for variation in the number of days spent during the last indoor period within production systems, relative age was included as a covariate in the following model:

y_ijkl_ = μ + α_i_ + β_j_ + αβ_ij_ + d_l_ + cov_ijkl_ + e_ijklm_

where μ = population mean, α_i_ = fixed effect of breed type, β_j_ = fixed effect of production system, αβ_ij_ = interaction between breed and production system, d_l_ = random effect of pen, cov_ijkl_ = covariate relative age for each individual, and e_ijklm_ = error term. Only differences between pure dairy (D) and crossbreed (C) were analysed, without assessing potential differences between the two dairy breeds. Means were compared pairwise using LSD_0_._05_-tests, adjusted by the method of Kenward and Roger [37], and denoted as significant at *p* < 0.05 and as a tendency for significance at 0.05 < *p* < 0.10.

## 3. Results

### 3.1. Technological Traits

The ultimate pH measurement after 24 h was higher for C steers compared to D steers (*p =* 0.030) and higher for H steers than for L steers (*p =* 0.001). The temperature after 24 h was significantly higher for H steers than for L steers (*p =* 0.025). Colour measurements showed differences in L* values between production systems, with meat from L steers appearing darker than meat from H steers (*p =* 0.003). There were no observed effects of breed type or production system on redness, yellowness, cooking loss, or WBSF (Table 3).

### 3.2. Sensory Properties

Breed type affected meat appearance and odour, as C steers exhibited a coarser fibre structure than D steers. The C steers also had a more sour/acidic odour and were perceived as less juicy. The meat from younger, more intensively reared H animals had a less visually intense red colour than the meat from L steers. An interaction between breed and production system (*p =* 0.045) was observed for metallic flavour, where CH had a higher score than CL (42.7 vs. 38.8) whereas DH and DL had similar scores (39.7 vs. 40.1) (Table 4).

### 3.3. The Fatty Acid Composition of Meat

There was an effect of both breed type and production system on the proportion of n-3 fatty acids, in which meat from C steers had a higher proportion than D steers and meat from L steers had a higher proportion than H steers. A significant effect of production system on total unsaturated fatty acids (MUFAs + PUFAs) was observed, where meat from L steers showed a higher proportion compared to H steers, due to the effect of the production system on the higher proportions of several individual fatty acids such as C18:1 t11, C18:2 n6, C18:3 n3, C18:2 c9 t11, C20:4 n6 (arachidonic acid, AA), and C20:5 n3 (eicosapentaenoic acid, EPA). An interaction between breed type and production system was found for C16:0 (*p =* 0.026), where the DL group showed a higher proportion than CL (27.2 vs. 25.5) while DH and CH showed the same (28.3 vs. 28.2). No effect of breed type was observed on the content of α-tocopherol and lutein, but the low feeding intensity resulted in higher α-tocopherol concentrations (Table 5).

## 4. Discussion

The primary aim of this study was to boost knowledge about the combination of conservation grazing on botanically diverse semi-natural pastures with the production of high-quality meat. The pasture consisted of approximately 20% dry, 60% mesic, and 20% wet areas and was mainly open but included small areas of mixed deciduous trees. The dominant plant species was *Deschampsia cespitosa* (tufted hairgrass), but *Festuca rubra* (red fescue) was also prominently present. In dry areas, *F. ovina* (sheep’s fescue), *D. flexuosa* (wavy hairgrass), *Nardus stricta* (matgrass), and several herb species were abundant. With the exception of *D. cespitosa* and *F. rubra*, herbs were prevalent in mesic areas, while *D. cespitosa* and *Cyperaceae* (sedges/rushes) were dominant in wet areas. The present research demonstrates differences in meat quality regarding the technological traits, sensory properties, and fatty acid profile between breed types (D and C) and production systems (H and L), which could be influenced by variations in slaughter age, carcass weight, and/or management practices.

There was an effect of breed on pH_24_, where C steers had comparatively higher values than D steers. Similarly, the production system affected pH_24_, whereby steers kept on high-intensity diets (H) had comparatively higher values than steers kept on low-intensity diets (L). However, it is important to note that all the pH values observed were within a low range, suggesting that glycogen depletion prior to slaughter was not a significant issue in this study. The amount of muscle glycogen decreased due to pre-slaughter stress [38] and the utilisation of energy in physical activity or emotional strain, increasing the ultimate pH of muscles [39]. Although there were statistically significant differences in pH_24_, the magnitude of these differences was small; therefore, it is unlikely that they would have a noticeable impact on eating quality. A very low pH could potentially affect water-holding capacity and cooking loss, but in this study, there were no significant differences in cooking loss between different production systems. Thus, based on the observed pH values, we do not anticipate any meaningful differences in eating quality.

The carcass characteristics in Table 1 indicate that crossbred steers (C) had a higher live weight at slaughter (682 kg vs. 640 kg) compared to dairy steers (D). These differences in live weight at slaughter could potentially influence the observed meat quality parameters, particularly in relation to pH and sensory traits. Similarly, steers from the H system displayed a lower live weight at slaughter than L steers (628 kg vs. 695 kg), aligning with their earlier slaughter age (21 vs. 28 months). These variations in live weight at slaughter and age may have contributed to differences in marbling, muscle structure, and fat composition, further reinforcing the observed effects on technological and sensory traits.

Meat from steers with a moderately high feeding intensity and one grazing season on semi-natural pastures (H) showed the effect of production system on temperature_24_ values. One reason for this could be the numerically higher fatness score of H steers compared to L steers (7.9 vs. 6.8; Table 1), as a low-intensity production system typically results in leaner, low-fat, and less-marbled carcasses. These results relate to the previous findings [40], whereby steers showed a higher post-slaughter temperature due to higher levels of subcutaneous fat protecting the carcass during the chilling process.

The consumer’s meat preference when purchasing is directly linked to its appearance and colour. There was an effect of production system on meat colour, whereby H steers showed higher L* values than L steers. Meanwhile, cooked meat from L steers was evaluated as having a pinker appearance by the analytical sensory panel (*p =* 0.008). Myoglobin (oxygen carrier haem pigment) and its oxidation state in muscle cells determines the meat colour [41,42] and can be modified in ruminants through production system or feeding regimes [43]. The higher L* values in H steers could be associated with lower values of haem iron due to slaughter occurring at an earlier age than the L steers (21 vs. 28 months). Myoglobin concentration increases rapidly until around 24 months of age, a phenomenon that which be linked to changes in muscle fibre types [41]. An increased slaughter age results in an increased proportion of red oxidative fibres in the muscles, which contain more myoglobin, leading to darker meat [44]. This observation aligns with previous studies that have highlighted the role of pasture-fed systems, where cattle typically have more physical activity and consumption of feed. These conditions stimulate the oxidative metabolism, leading to a greater development of type I muscle fibres and, consequently, darker meat compared to a grain-fed intensive system [45,46]. Additionally, specific feed ingredients can stimulate oxidative metabolism, as demonstrated by research on the genetic and nutritional manipulation of meat quality [46]. Our results are in accordance with another important aspect, which is that animals raised in pasture-based production systems are often older and, hence, have a darker meat compared to those raised in grain-fed intensive production systems [47]. These factors collectively influence the muscle fibre composition and overall meat quality, thus reinforcing the complex relationship between diet, exercise, and age in beef production. Therefore, age differences between steers of two production systems may be important in explaining why the sensory panel evaluated the meat appearance of H steers as less pinkish compared to that of the L steers.

Regarding meat quality, tenderness is usually the highest-ranked beef quality attribute [48], and measurements of WBSF reflect the tenderness of meat. Different factors including ultimate pH, duration of proteolysis, intramuscular fat, and length of sarcomere are correlated to the tenderness of meat [49]. There was no effect of either breed type or production system on WBSF values, indicating that the tenderness measured instrumentally was not influenced by these variables. These findings were further supported by sensory evaluations, which also found no differences in tenderness.

The carcass conformation score was higher in crossbred steers than in dairy steers (5.7 vs. 4.0; Table 1), which is expected due to the genetic contribution of beef breeds. This trait is closely linked to muscling and overall meat yield, which may partially explain differences in sensory perceptions of fibre structure between breed types. Marbling (intramuscular fat content) is another major factor that can influence the sensory properties of meat [50]. However, the intake of fibrous diets can negatively affect both marbling and tenderness, particularly when animals are on these diets for longer periods. This is not simply due to a lighter body weight but rather the reduced growth rate associated with high-fibre low-starch diets, which limits fat deposition and, thus, marbling [51]. When comparing diets over a specific period, animals consuming diets that are more fibrous generally exhibit slower growth and lower energy intake, leading to reduced marbling compared to those on higher-starch diets that promote faster growth and greater fat accumulation. As a result, the lower energy density of fibrous diets hinders the development of intramuscular fat, affecting both marbling and the tenderness of the meat.

The C steers in this study exhibited a coarser fibre structure compared to the purebred D steers, which could be linked to differences in the types of muscle fibres or the distribution of intramuscular fat between the fibres. Coarser fibres are frequently associated with a higher proportion of type IIB (slow-twitch) muscle fibres, which are typically larger in diameter and have a more pronounced structure [52]. Additionally, a tendency to lower marbling content in C steers (*p =* 0.054; [20]) may have contributed to this coarser appearance, as less intramuscular fat between muscle fibres can make the fibres appear more distinct and less smooth [53]. Previous studies found a link between higher marbling with more desirable sensory properties [54,55], i.e., tenderness and juiciness. The lubrication effect of marbled meat stimulates salivation, providing a sense of increased juiciness and flavour while chewing. Similarly, a higher percentage of marbling results in low-density steak (which requires less resistance to chew) and contributes to a more tender eating experience [56]. The crossbred steers in this study also exhibited a more pronounced acidic odour compared to D steers. However, the perception of an acidic odour in beef is complex and can be influenced by a variety of factors. While marbling typically enhances the juiciness and overall flavour profile of beef, the distinct acidic odour observed in the C steers may have been a combination of diet, breed type, and post-mortem handling as opposed to marbling alone [57]. Moreover, the higher pH_24_ values observed in C steers could have contributed to an environment that supported the growth of lactic acid bacteria during ageing, which may have enhanced the acidic odour [58,59]. Further, crossbreeding with beef cattle could have influenced the muscle fibre types, potentially affecting the metabolic pathways and leading to the production of certain compounds which could have also contributed to this distinct odour [60]. Overall, the differences observed in the major sensory properties between the breed types and production systems were minimal and may not be large enough to influence consumer preferences. It can therefore be concluded that C steers were comparable to D steers and L steers were comparable to H steers in terms of sensory quality.

Fatty acid composition is an important indicator of the nutritional value of meat, and it also affects shelf life and flavour [61]. There was an effect of production system on total unsaturated fatty acids and n-3 fatty acids, whereby L steers had higher values than H steers for both. Unsaturated fatty acids are considered beneficial for human health, particularly PUFA, with an appropriate ratio of n-6/n-3, and can play a favourable role in the prevention of human diseases such as heart disease, obesity, and cancer [62]. Contrastingly, H steers showed higher values for total saturated fatty acids, which are generally considered unhealthy and potentially harmful due to hyper-cholesterolimic properties of SFA, which can cause atherosclerosis and increase the risk of cardiovascular disease [63]. There was also an effect of breed type and production system on n-3 proportion, whereby C and L steers had higher proportions than D and H steers, respectively. Diet composition is a major factor affecting fatty acid composition in beef production. Ruminants naturally consume diverse diets, including fresh grass, preserved forage, and concentrates, each of which can lead to a significant variation in fat and PUFA content. Fresh grass is typically higher in PUFA, while concentrates can be higher in fat but lower in PUFA [64]. The concentration of C18:3 n3 in meat typically increases when animals are fed grass and forage. Another important factor that influences differences in fatty acid profiles is the breed’s age at slaughter maturity. For example, late-maturing breeds such as Charolais often exhibit different fat deposition patterns compared to early-maturing breeds [54]. Since fat content plays a crucial role in determining the fatty acid composition of meat, these breed-related differences significantly impact the overall fatty acid profile [27,65]. Similarly, late-maturing breeds, which are leaner at slaughter, usually have a higher proportion of PUFA within their meat compared to early-maturing breeds [64]. Further, there was an effect of production system on n-6/n-3 ratio, whereby L steers showed higher values than H steers. This difference was likely due to the increased levels of n-3 fatty acids in L steers, which had been reared on a low-intensity feed system with grazing on semi-natural pastures. Higher n-3 levels would typically decrease the n-6/n-3 ratio, which is a favourable outcome. However, in this case, both ratios were relatively low. An additional explanation for the observed higher ratio in L steers could be a lower de novo synthesis of fatty acids in these animals compared to H steers, which likely resulted in a lower content of SFA and MUFA, particularly C18:1. This reduced synthesis could have further influenced the overall fatty acid profile, leading to the observed differences in the n-6/n-3 ratio [66]. The World Health Organization recommended the n-6/n-3 ratio in a healthy diet to be 4:1 or below [67], and this is associated with a lower probability of both cardiovascular disease and cancer [68]. In the present study, the n-6/n-3 ratio values observed were from 1.24 to 1.38, which did not exceed the maximum recommended levels, indicating it as healthy meat, in line with previous finding whereby silage-fed steers consistently showed an n-6/n-3 ratio around 1.3 [64]. In general, these results are in accordance with previous findings whereby pasture-fed beef animals showed more PUFAs, especially the proportion of n-3 [69,70].

Overall, beef production with two grazing summers on semi-natural pastures resulted in a higher-quality meat with a more favourable fatty acid profile, including increased levels of unsaturated and n-3 fatty acids. However, given that the fat content is 2% or below, this lean product may have limited impact on the human diet in terms of fatty acid intake. Nonetheless, these attributes could still add value to pasture-raised beef through marketing strategies or direct sales from farmers to consumers, emphasising the benefits of sustainable food production, biodiversity conservation, and the preservation of cultural heritage. Furthermore, previous findings of the same project [20,22] found that a production system with semi-natural pastures showed economically better results for farmers, alongside the production of heavier and higher-quality carcasses using beef breed sires with dairy cows. The fibre structure appearance and acidic odour of the C steers could potentially be improved through changes in production system, systematic strategies for crossbreeding, and, most importantly, pre- and post-slaughter handling.

## 5. Conclusions

Overall, few minor differences in technological traits, sensory attributes, and fatty acid composition were observed between the breed types and production systems. This supports the idea that conservation grazing of biodiverse semi-natural pastures can produce uniform meat quality, adaptable to the specific conditions of the farm. The low-intensity grazing system (L), with two summers on biodiverse pastures, produced darker meat with a higher proportion of unsaturated and n-3 fatty acids, suggesting benefits for meat nutritional value. In contrast, the high-intensity system (H) resulted in lighter meat with higher saturated fatty acids. Breed type also influenced meat quality, with crossbred (C) steers showing higher n-3 and n-6 fatty acids, a coarser fibre structure, and a more acidic odour compared to dairy (D) steers. These differences could be attributed to both breed characteristics and the production systems. However, the overall uniformity in quality across systems suggests that this approach is most valuable when aligned with broader sustainability goals, such as biodiversity conservation and cultural heritage preservation, rather than solely for enhancing meat quality.

## Figures and Tables

**Table 1:** Carcass characteristics of the two breed types (dairy, D; dairy × beef crossbred, C) in two production systems (moderately high indoor feed intensity and 21 months of slaughter age, H, vs. low indoor feed intensity and 28 months of slaughter age, L; all grazing on semi-natural pastures in the summer).

Carcass Traits	Breed Type		Production System	
n	D 29	C 32	H 30	L 31
LWG ^a^ weaning–slaughter, kg/day	0.84	0.86	0.94	0.77
Live weight at slaughter, kg	640	682	628	695
Carcass weight, kg	294	335	299	329
Dressing (%)	45.8	49.1	47.6	47.3
Conformation ^b^	4.0	5.7	4.6	5.1
Fatness ^c^	7.2	7.4	7.9	6.8
Marbling ^d^	2.0	1.5	1.7	1.8

^a^ LWG = live weight gain; ^b^ EUROP system: 4 = O−, 5 = O, and 6 = O+; ^c^ EUROP system: 6 = 2+, 7 = 3−, and 8 = 3; ^d^ visually determined in the M. longissimus lumborum between the 10th and 11th ribs on a scale from 1 = lean to 5 = fat.

**Table 2:** Definitions of the sensory attributes tested.

Category	Attribute	Definition
**Odour**	Iron/blood	A metallic scent associated with undercooked meat
	Acidic	Acidic scent
	Fatty	Sensation associated with the smell of cooked tallow
	Barny	Odours associated with faeces, animals, and stables
	Milky	Milky scent, associated with dairy cattle
**Appearance**	Fibre structure	Ocular assessment of coarseness of muscle fibres, from fine to coarse
	Pink colour	Intensity of pink in the centre of the sample, ranging from weak pink to red
	Connective tissue appearance	Degree of white strikes in the meat
**Texture**	Resistance	Force required to cut through the meat using a table knife three times across the fibres
	Tenderness	Degree of tenderness, from low to high, after chewing three times with the molars
	Crumbliness	Disruption, from low to high, after chewing six times with the molars
	Juiciness	Sensation caused by meat with higher levels of juices, from low to high, after chewing six times with the molars
**Taste**	Umami	Taste elicited by monosodium glutamate
	Sour	Taste elicited by acids
	Salt	Taste elicited by sodium chloride
**Flavour**	Metallic flavour	Flavour associated with various metal flavours
	Barny flavour	Flavour associated with faeces, animals, and stables
	Game flavour	Taste associated with wild game meat

**Table 3:** Effect on technological meat quality traits from both breed types (dairy, D, vs. dairy × beef crossbreds, C) and production systems (moderately high indoor feed intensity and 21 months of slaughter age, H, vs. low indoor feed intensity and 28 months of slaughter age, L; all grazing on semi-natural pastures in the summer). Bold *p*-values are significant.

Technological Traits	Breed Type		Production System			*p*-Value	
n	D 29	C 32	H 30	L 31	S.e	B	P
pH_24_	5.30	5.36	5.43	5.22	0.02	**0.030**	**0.000**
Temperature_24_	6.74	6.90	7.20	6.43	0.11	0.591	**0.025**
Lightness (L*)	37.3	38.1	39.0	36.3	0.50	0.289	**0.003**
Redness (a*)	24.6	24.6	24.1	25.1	0.40	0.940	0.132
Yellowness (b*)	12.8	13.0	13.1	12.7	0.31	0.537	0.336
Cooking loss	12.0	12.2	12.3	11.9	0.28	0.703	0.322
WBSF	57.6	53.2	54.5	56.3	2.93	0.315	0.666

S.e = standard error; WBSF = Warner–Bratzler shear force; B = breed type; and P = production system. No B × P interaction was found.

**Table 4:** Effects on sensory properties from both breed types (dairy, D, vs. dairy x beef crossbreds, C) and production systems (moderately high indoor feed intensity and 21 months of slaughter age (H) vs. low indoor feed intensity and 28 months of slaughter age (L), all grazing on semi-natural pastures in the summer). Bold *p*-values are significant.

Category	Attribute	Breed Type		Production System			*p*-Value	
	n	D 29	C 32	H 30	L 31	S.e	B	P
Odour	Iron/Blood	30.1	31.2	30.2	31.1	0.71	0.234	0.448
	Acidic	25.0	27.1	26.4	25.7	0.82	**0.040**	0.554
	Fatty	25.7	24.8	25.3	25.1	0.66	0.255	0.842
	Barny	29.2	29.3	29.8	28.6	0.85	0.959	0.361
	Milky	24.4	25.3	25.2	24.4	0.70	0.279	0.459
Appearance	Fibre structure	35.1	38.8	36.4	37.5	1.21	**0.022**	0.547
	Pink colour	52.4	47.8	45.9	54.2	1.99	0.080	**0.008**
	Connective tissue	32.3	30.3	32.8	29.8	2.29	0.546	0.434
Texture	Resistance *	33.7	39.6	34.8	38.5	5.19	0.440	0.654
	Tenderness	49.4	46.3	51.0	44.7	0.36	0.759	0.597
	Crumbliness	39.9	45.7	44.3	41.3	3.64	0.300	0.611
	Juiciness	47.0	43.9	45.8	45.1	0.19	0.344	0.851
Taste	Umami	38.3	36.2	38.2	36.4	1.20	0.083	0.354
	Sour	19.0	21.7	21.1	19.6	1.67	0.225	0.538
	Salt	30.2	28.5	29.6	29.2	1.84	0.463	0.880
Flavour	Metallic	39.9	40.7	41.2	39.4	0.80	0.392	0.146
	Barny/Stable	30.5	30.9	31.9	29.4	1.26	0.730	0.111
	Game	22.4	19.8	23.9	18.3	2.00	0.301	0.076

S.e = standard error; * = cutting by hand; B = breed type; and P = production system. The interaction B × P for metallic (flavour) was *p* = 0.045.

**Table 5:** Effect on fat content (g/100 g tissue), fatty acid composition (g/100 g FA), and vitamin concentration from both breed types (dairy, D, vs. dairy x beef crossbreds, C) and production systems (moderately high indoor feed intensity and 21 months of slaughter age (H) vs. low indoor feed intensity and 28 months of slaughter age (L), all grazing on semi-natural pastures in the summer). Bold *p*-values are significant.

Fatty Acids and Vitamins	Breed Type		Production System			*p*-Value	
n	D 29	C 32	H 30	L 31	S.e	B	P
Fat	2.10	1.37	1.54	1.92	0.15	**0.005**	0.113
C14:0	2.01	1.64	1.97	1.68	0.07	**0.001**	**0.012**
C14:1	0.59	0.44	0.50	0.53	0.04	**0.019**	0.648
C15:0	0.39	0.43	0.43	0.38	0.01	0.072	**0.025**
C16:0	27.7	26.8	28.2	26.4	0.28	**0.019**	**0.001**
C16:1 n9	0.23	0.28	0.24	0.27	0.01	**0.003**	**0.020**
C16:1 n7	3.98	3.10	3.41	3.66	0.20	**0.012**	0.392
C17:1	1.04	1.05	1.11	0.98	0.03	0.912	**0.018**
C18:0	12.1	12.9	13.0	12.1	0.39	0.172	0.154
C18:1 n9	40.0	38.0	39.8	38.2	0.54	**0.034**	**0.064**
C18:1 t11	2.35	2.24	1.96	2.64	0.06	0.219	**<0.0001**
C18:1 n7	0.18	0.12	0.14	0.16	0.02	**0.024**	0.607
C18:2 n6	2.69	3.90	2.46	4.13	0.25	**0.012**	**0.001**
C18:3 n3	1.31	1.94	1.34	1.91	0.10	**0.004**	**0.005**
C18:3 n6	0.59	0.90	0.79	0.70	0.06	**0.005**	0.291
C18:2 c9 t11	0.23	0.22	0.16	0.29	0.01	0.700	**<0.0001**
C18:2 c12 t10	0.02	0.03	0.02	0.02	0.00	**0.018**	0.202
C20:0	0.09	0.10	0.09	0.10	0.01	0.192	0.436
C20:1 n9	0.15	0.12	0.14	0.13	0.01	**0.005**	0.278
C20:3 n6	0.28	0.39	0.25	0.43	0.03	**0.016**	**0.001**
C20:4 n6 (AA)	1.34	1.74	1.22	1.87	0.11	**0.018**	**0.003**
C20:5 n3 (EPA)	0.87	1.17	0.89	1.15	0.08	**0.022**	**0.044**
C22:5 n6	0.11	0.14	0.11	0.15	0.01	**0.017**	**0.014**
C22:5 n3 (DPA)	1.17	1.64	1.26	1.56	0.09	**0.004**	**0.038**
C22:6 n3 (DHA)	0.16	0.22	0.17	0.21	0.01	**0.015**	0.098
Total n3	3.80	5.36	3.90	5.26	0.30	**0.005**	**0.013**
Total n6	5.01	7.08	4.82	7.27	0.44	**0.009**	**0.003**
Ratio n6/n3	1.31	1.32	1.24	1.38	0.01	0.978	**<0.001**
Total SFA	42.3	41.7	43.7	40.6	0.57	0.648	**0.005**
Total USFA	57.4	57.7	56.0	59.0	0.57	0.702	**0.006**
SFA:USFA	0.74	0.73	0.78	0.69	0.02	0.681	**0.006**
α-tocopherol	3.08	2.88	2.49	3.46	0.17	0.456	**0.006**
Lutein	0.06	0.05	0.05	0.06	0.01	0.610	0.345

S.e = standard error; B = breed type; and P = production system. The interaction B × P for C16:0 was *p =* 0.026.

## Data Availability

Dataset available upon request from the authors.
